# Evaluation of the VETSCAN IMAGYST: an in-clinic canine and feline fecal parasite detection system integrated with a deep learning algorithm

**DOI:** 10.1186/s13071-020-04215-x

**Published:** 2020-07-11

**Authors:** Yoko Nagamori, Ruth Hall Sedlak, Andrew DeRosa, Aleah Pullins, Travis Cree, Michael Loenser, Benjamin S. Larson, Richard Boyd Smith, Richard Goldstein

**Affiliations:** 1 Department of Veterinary Pathobiology, College of Veterinary Medicine, Oklahoma State University, Stillwater, OK 74078 USA; 2 Zoetis, Veterinary Medicine Research and Development, 333 Portage St, Kalamazoo, MI 49007 USA; 3 Zoetis, Global Diagnostics, 10 Sylvan Way, Parsippany, NJ 07054 USA; 4 Techcyte Inc., 384 S 400 W #125, Lindon, UT 84042 USA

**Keywords:** Deep learning, Fecal egg identification, Artificial intelligence, Veterinary parasitology diagnostic

## Abstract

**Background:**

Fecal examination is an important component of routine companion animal wellness exams. Sensitivity and specificity of fecal examinations, however, are influenced by sample preparation methodologies and the level of training and experience of personnel who read fecal slides. The VETSCAN IMAGYST system consists of three components: a sample preparation device, a commercially available scanner, and an analysis software. The VETSCAN IMAGYST automated scanner and cloud-based, deep learning algorithm, locates, classifies, and identifies parasite eggs found on fecal microscopic slides. The main study objectives were (i) to qualitatively evaluate the capabilities of the VETSCAN IMAGYST screening system and (ii) to assess and compare the performance of the VETSCAN IMAGYST fecal preparation methods to conventional fecal flotation techniques.

**Methods:**

To assess the capabilities of VETSCAN IMAGYST screening components, fecal slides were prepared by the VETSCAN IMAGYST centrifugal and passive flotation techniques with 100 pre-screened fecal samples collected from dogs and cats and examined by both the algorithm and parasitologists. To determine the diagnostic sensitivity and specificity of the VETSCAN IMAGYST sample preparation techniques, fecal flotation slides were prepared by four different techniques (VETSCAN IMAGYST centrifugal and passive flotations, conventional centrifugal flotation, and passive flotation using OVASSAY® Plus) and examined by parasitologists. Additionally, required sample preparation and scanning times were estimated on a subset of samples to evaluate VETSCAN IMAGYST ease-of-use.

**Results:**

The algorithm performance of the VETSCAN IMAGYST closely matched that of the parasitologists, with Pearsonʼs correlation coefficient (*r*) ranging from 0.83–0.99 across four taxa of parasites, *Ancylostoma*, *Toxocara*, *Trichuris* and Taeniidae. Both VETSCAN IMAGYST centrifugal and passive flotation methods correlated well with conventional preparation methods on all targeted parasites (diagnostic sensitivity of 75.8–100%, specificity of 91.8–100%, qualitative agreement between methods of 93.8–94.5%). Sample preparation, slide scan and image analysis were completed within 10–14 min by VETSCAN IMAGYST centrifugal and passive flotations, respectively.

**Conclusions:**

The VETSCAN IMAGYST scanning system with the VETSCAN IMAGYST sample preparation methods demonstrated a qualitative match in comparison to the results of parasitologists’ examinations with conventional fecal flotation techniques. The VETSCAN IMAGYST is an easy-to-use, next generation qualitative and possibly quantitative diagnostic platform that brings expert clinical results into the hands of veterinary clinics.
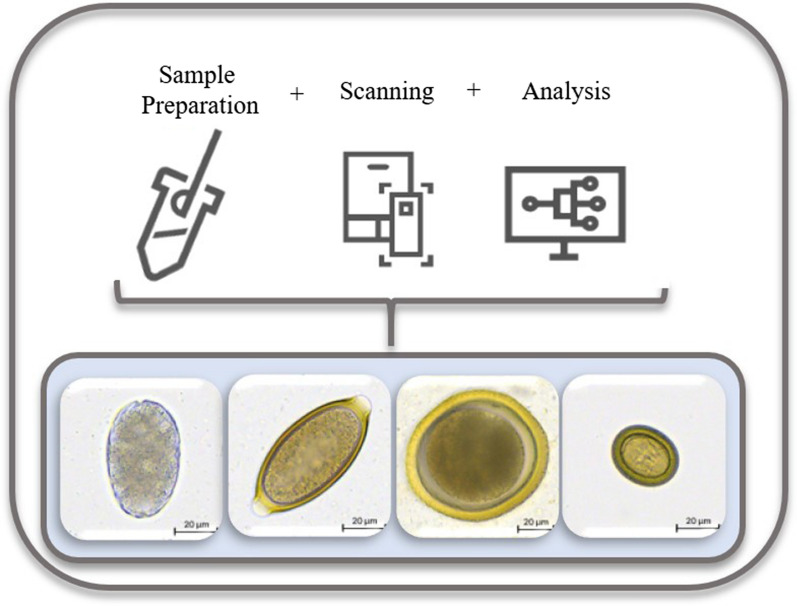

## Background

Fecal screening for parasitic infections in dogs and cats is an important part of wellness examinations. Standard diagnostic tests performed in most veterinary practices involve a passive or centrifugal fecal flotation followed by microscopical examination, looking for various parasitic elements, such as eggs, oocysts, cysts, larvae, and occasionally trophozoites. However, diagnostic accuracy and sensitivity of fecal examinations vary widely depending on the level of training and experience of the personnel who read slides as well as the fecal preparation methods utilized at clinics [1–7]. Although microscopical examination of fecal slides should be done carefully and thoroughly by veterinarians or trained technicians, the assignment is often conducted rapidly by staff with little experience or emphasis on the significance of the examination. Previous studies demonstrated that centrifugation increased sensitivity of fecal examinations compared to passive flotation, especially for detection of *Trichuris vulpis* eggs and *Giardia* cysts [2, 4, 8]; however, many practitioners still prefer using a passive flotation method due to its convenience. Gates & Nolan [6] suggested that fecal flotation examinations performed in private practice could be missing up to half of infected dogs because of either technician error or inherent limitations to the passive flotation technique.

**Fig. 1:**
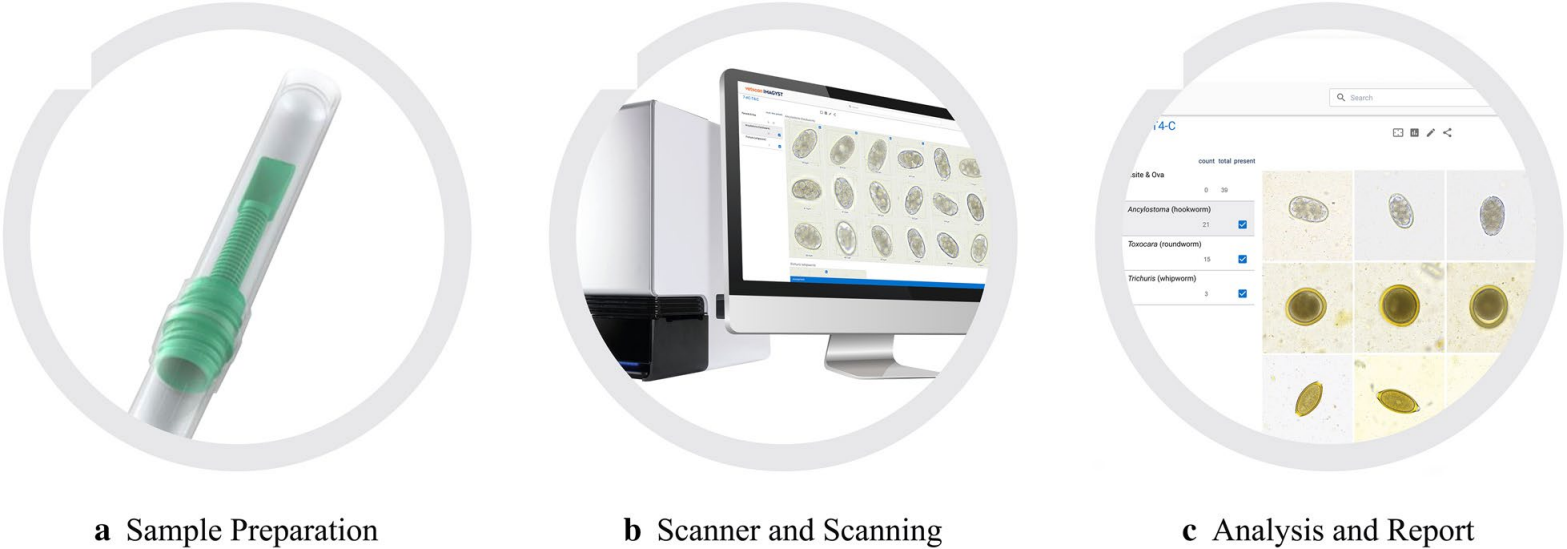
VETSCAN IMAGYST fecal flotation uses a simple, proprietary sample preparation method to create slides for digitization and automatic uploading to the cloud for analysis by deep learning algorithms for common intestinal parasite eggs

The VETSCAN IMAGYST system was developed to provide a simpler, easier, and more systematized fecal examination, which is less influenced by different fecal preparation methodologies or level/experience of an examiner. It is composed of three elements: a sample preparation device; an automated commercially available microscopic scanner; and data analysis by deep neural networks (Fig. 1). The primary objective of this study was to qualitatively evaluate the diagnostic performance of the VETSCAN IMAGYST screening elements to correctly identify helminth eggs in feces of naturally infected dogs and cats, compared to a manual identification by a parasitologist. A secondary objective was to compare the performance of the VETSCAN IMAGYST sample preparation methods to standard reference methods of centrifugal and passive fecal flotation. As part of the performance assessment, the time for sample preparation, scan, and data analysis was monitored and recorded to evaluate the usability of VETSCAN IMAGYST screening system with the VETSCAN IMAGYST centrifugal and passive fecal flotation techniques.

## Methods

### Fecal sample collection and pre-screening

Fecal samples from client-owned and shelter dogs and cats submitted to the veterinary parasitology diagnostic laboratory at Oklahoma Animal Disease Diagnostic Laboratory of Oklahoma State University were pre-screened by the Wisconsin fecal egg counting test [8] with Sheather’s sugar solution (specific gravity of 1.25) or 33% zinc sulfate solution (specific gravity of 1.18). The performance assessment utilized fecal samples weighing a minimum of 8 g confirmed positive for *Ancylostoma*, *Toxocara*, *Trichuris*, and/or taeniid eggs, or confirmed negative for parasite eggs for use as negative controls. Eggs of canine *Ancylostoma* were differentiated from those of *Uncinaria stenocephala* and other strongylids based on morphological features and size [8]. A minimum of 10 fecal samples for each targeted parasite were prepared, and a total of 100 fecal samples were included for this study. Of the 100 samples, 84 were from dogs and 16 were from cats with 44 samples identified as a co-infection with two or more intestinal parasites. To maintain morphological integrity of the parasite eggs, all samples were preserved at 4 °C until the study. Sixty-two samples were analyzed by 2 weeks post-sampling (without addition of preservation solution) and 38 samples were fixed with 10% formalin solution.

### VETSCAN IMAGYST scanning and analyzing systems

For a scanning component of the VETSCAN IMAGYST system, the Motic EasyScan One® digital slide scanner (Motic, Kowloon Bay, Hong Kong) was utilized. This automated scanner read fecal slides with a 20 magnification 0.75 numerical aperture (N/A) apochromatic and flat field correction (Plan Apo) objective, providing 40× effective resolution. Captured images were then automatically uploaded, processed, and analyzed in the cloud using the VETSCAN IMAGYST analysis software (Zoetis Inc., Parsipanny Troy Hills, New Jersey, USA). A deep learning object detection network based on Single Shot MultiBox Detector (SSD) [9] with Inception v2 [10] backbone was used for localization and classification. Briefly, the scans were cropped into tiles that were passed into the classifier which simultaneously predicted and classified multiple bounding boxes on each crop with confidence, analyzing for whether there were objects of interest (i.e. *Ancylostoma*, *Toxocara*, *Trichuris* and taeniid eggs). Once analysis on the image of the fecal slide was completed, a result with image(s) of targeted parasite eggs became available for review on the VETSCAN IMAGYST platform in a web browser. Although the VETSCAN IMAGYST analysis software has an option enabling counting, this feature was not evaluated in this study since our main purpose was to better understand the qualitative capability of the algorithm to recognize parasite eggs.

### VETSCAN IMAGYST algorithm assessment

**Fig. 2:**
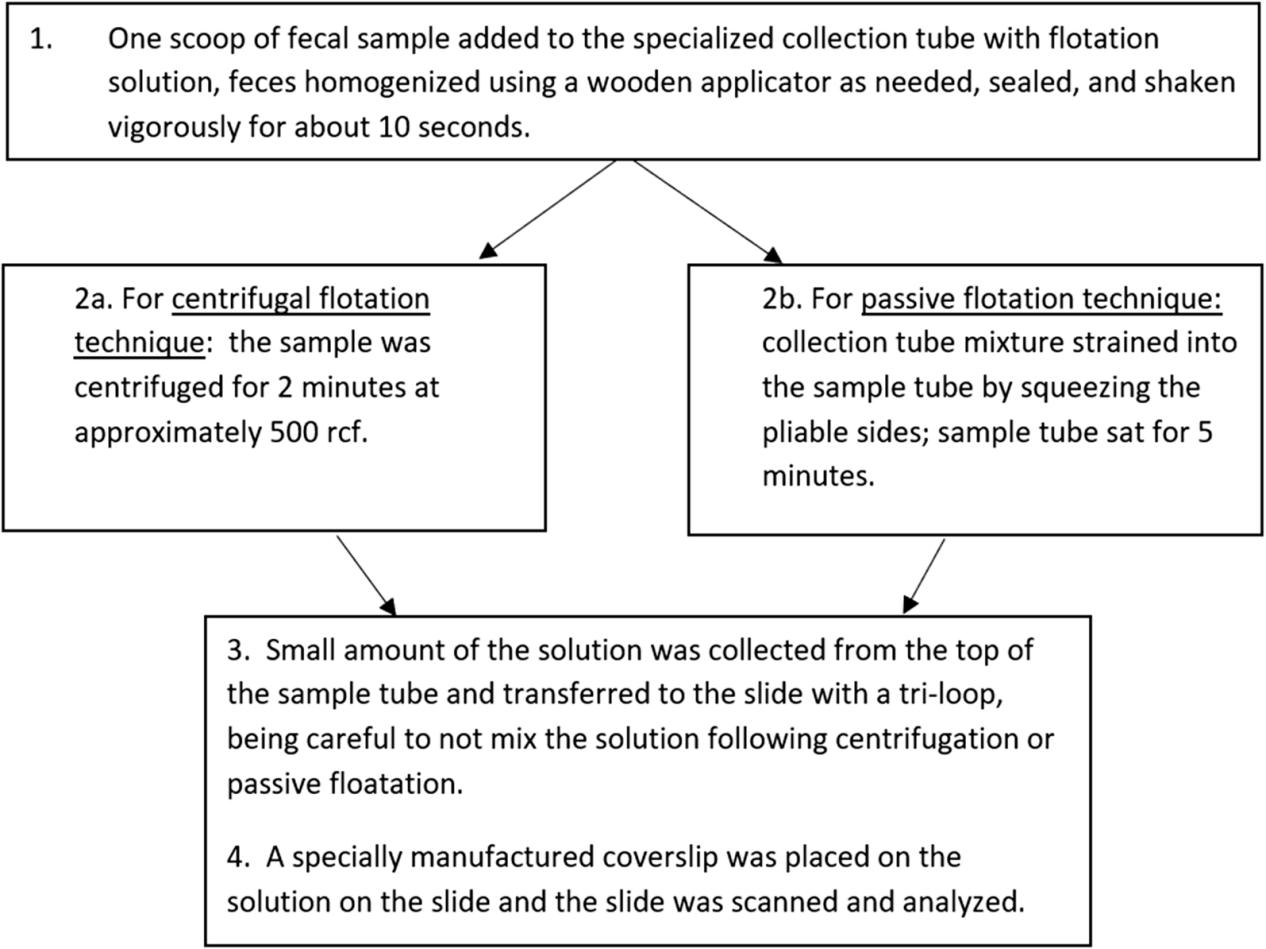
Summary of VETSCAN IMAGYST centrifugal flotation and passive flotation sample preparation methods

To qualitatively evaluate the ability of the scanning and analyzing components of the VETSCAN IMAGYST system in identifying eggs of the targeted parasites, slides were prepared with the VETSCAN IMAGYST centrifugal flotation and VETSCAN IMAGYST passive flotation sample preparation techniques on each pre-screened fecal sample (Fig. 2). Pre-screened fecal samples were labeled 1–100 and examined randomly out of order. Apacor mini Parasep® SF (Apacor Ltd., Wokingham, UK) was specifically re-designed and produced as the VETSCAN IMAGYST fecal preparation device and utilized to perform the VETSCAN IMAGYST centrifugal and passive flotation methods. The device consisted of two tubes, the sample tube with a sample scoop, and the collection tube containing sugar flotation solution (specific gravity of 1.25). Briefly, the cap of the collection tube was unscrewed, one scoop of fecal sample (1 g of feces) was added to the collection tube, and feces with flotation solution was homogenized well using a wooden applicator or stick stirrer as needed. After the sample and collection tubes were tightly sealed, the collection tube was shaken vigorously for about 10 s. For centrifugal flotation technique, the collection tube was placed in a centrifuge for 2 min at 500 *rcf*. For the passive flotation technique, the mixture solution in the collection tube was strained into the sample tube by squeezing the pliable sides, and the solution in the sample tube was incubated at room temperature for 5 min. Using a tri-transfer loop, a small amount of the solution was collected from the top of the tube and placed on a slide. A specially manufactured coverslip (Apacor Ltd., Wokingham, UK) was placed on the solution on the slide, and the slide was placed in a slide tray. The tray was then inserted into an automated microscopic scanner. The digital scanned image was automatically uploaded to the cloud for analysis and generation of results.

The total time for sample preparation, scanning, analysis and results generation was monitored and recorded to evaluate the usability of the system. Two technicians performed the VETSCAN IMAGYST fecal preparation techniques and allowed the VETSCAN IMAGYST system to screen and analyze the slides. Followed by the VETSCAN IMAGYST examinations, three experienced diagnostic parasitologists read all the slides microscopically using 100×, 200×, and 400× magnifications. Parasites were identified to genus/family based on egg morphology and recorded [8]. Quantitative counts of eggs on the entire slide were performed by the parasitologists, up to 50 eggs per each targeted parasite, while the algorithm provided counts regardless of egg burden. For slides with > 50 eggs, samples were characterized as medium (> 50, ≤ 250) or high (> 250). Results from the VETSCAN IMAGYST system and microscopical examinations by parasitologists were compared and analyzed statistically.

### Sample preparation method assessment

Performance (sensitivity and specificity) of the VETSCAN IMAGYST centrifugal and VETSCAN IMAGYST passive flotation techniques were assessed by comparing to the conventional centrifugal and passive flotation methods using visual microscopy. With each fecal sample, slides were prepared by four different sample preparation techniques: (i) VETSCAN IMAGYST centrifugal flotation; (ii) VETSCAN IMAGYST passive flotation; (iii) conventional centrifugal flotation; and (iv) passive flotation. Slides representing the VETSCAN IMAGYST flotation techniques were prepared by two technicians as described previously. For the reference centrifugal fecal flotation technique, approximately 1 g of feces was weighed and suspended in Sheather’s sugar solution (specific gravity of 1.25), strained with a piece of cheesecloth to remove debris, and placed in a 15 ml centrifuge tube. The tube was filled with flotation solution until a convex meniscus was formed and a coverslip was placed on the top. The samples were centrifuged in a Centra CL2 centrifuge (Thermo Fisher Scientific, Waltham, Massachusetts, USA) at approximately 440 *rcf* for 5 min. The coverslip was removed and placed on a glass slide for microscopic examination [8]. The passive fecal flotation test was performed using the OVASSAY® Plus Kit Fecal Flotation Device (Zoetis Inc.) with 33% zinc sulfate solution (specific gravity of 1.18), following the manufacturer’s instructions [11]. Several student workers prepared the fecal slides with conventional fecal preparation methods, and all slides were microscopically examined by three parasitologists as described previously.

### Statistical analysis

Samples were considered positive if any eggs were observed. Two-by-two (2 × 2) tables were calculated. Sensitivity and specificity together with 95% Jeffreys’ confidence interval estimates were calculated. Because the microscopy estimates by the parasitologists were quantitative at 50 eggs or less on each slide, scatter plots were created and Pearsonʼs correlation coefficient (*r*) calculated limiting all methods at 50 eggs or less. Any results larger than 50 were removed from the scatter plots and correlation coefficients. SAS version 9.4M6 (SAS Institute Inc.; Cary, North Carolina, USA) was used for statistical analysis.

## Results

### Algorithm performance

**Fig. 3:**
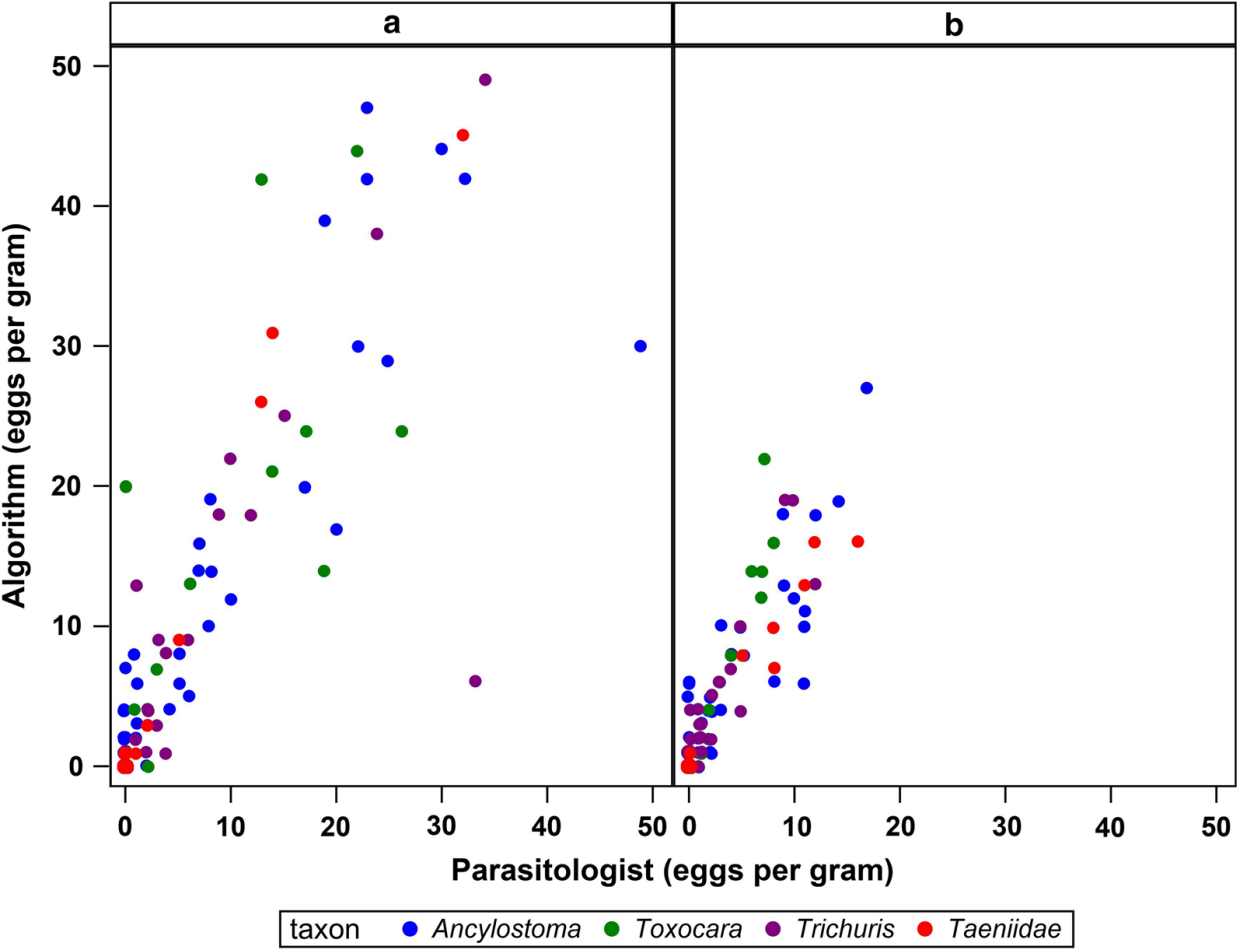
Scatter plot of VETSCAN IMAGYST centrifugal flotation method (**a**) and VETSCAN IMAGYST passive flotation method (**b**) read by a parasitologist (x-axis) *versus* read by the VETSCAN IMAGYST algorithm (y-axis)

**Fig. 4:**
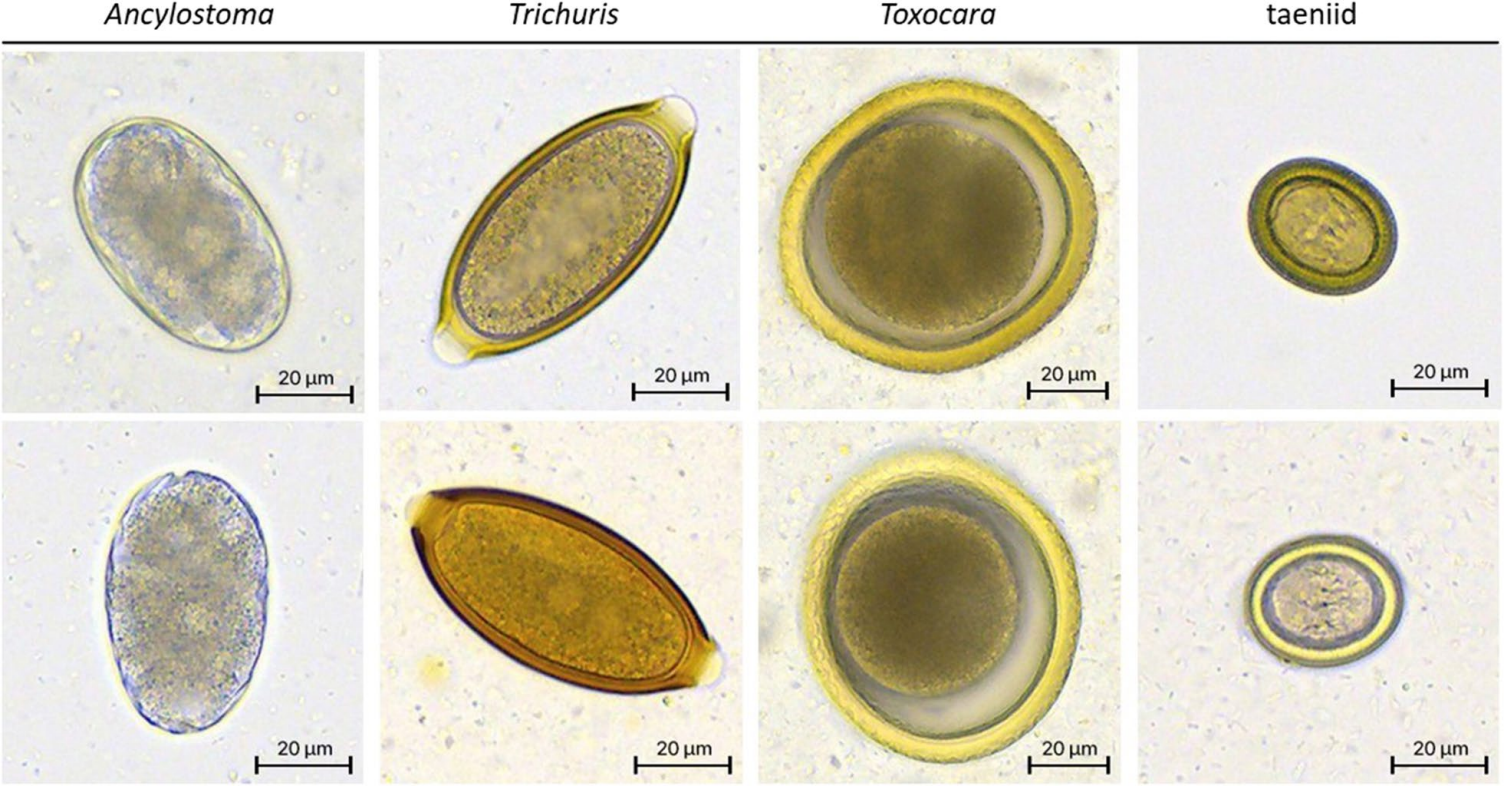
VETSCAN IMAGYST images of individual fecal parasite eggs. Each column presents a representative image of each taxon

The ability of the VETSCAN IMAGYST system to accurately identify eggs of targeted canine and feline parasites is driven by the integrated deep learning object detection algorithm that reads scanned slide images. The algorithm’s performance was assessed by comparing parasitologists’ results to the algorithm’s results on the same slide prepared either by VETSCAN IMAGYST centrifugal flotation or VETSCAN IMAGYST passive flotation (Fig. 3, Table 1). For both slide preparation methods, the VETSCAN IMAGYST diagnostic result closely matched that of the parasitologists, with a Pearson’s correlation coefficient (*r*) ranging between 0.83–0.99 (Fig. 3, Table 1). Images representative of the four targeted parasite eggs from each slide assessed by the VETSCAN IMAGYST algorithm are shown in Fig. 4.

**Table 1:** Pearson’s correlation coefficient of VETSCAN IMAGYST centrifugal flotation method and VETSCAN IMAGYST passive flotation method read by a parasitologist *versus* read by the VETSCAN IMAGYST algorithm

	*Ancylostoma*	*Trichuris*	*Toxocara*	Taeniidae
Centrifugal flotation	0.90	0.83	0.86	0.98
Passive flotation	0.95	0.97	0.84	0.99

### Sample preparation performance

The VETSCAN IMAGYST system consists not only of an image analysis driven by a deep learning algorithm, but also a specialized fecal preparation device that uses the centrifugal flotation or passive flotation method. The performance of this device used for both passive flotation and centrifugal flotation (test methods) was compared to the performance of OVASSAY passive flotation (reference method) (Table 2) and conventional centrifugal flotation (reference method) (Table 3). The egg recovery of all methods was assessed through diagnostic sensitivity and specificity. Both VETSCAN IMAGYST passive and centrifugal flotation methods correlated well with conventional preparation methods across the parasite taxa (Tables 2, 3). Agreement (defined as the number of true positives and true negatives divided by the total number of samples) between the two passive flotation techniques was 93.8% across the four targeted parasites. In this evaluation, VETSCAN IMAGYST passive flotation detected 17 samples that OVASSAY did not; OVASSAY detected 9 that VETSCAN IMAGYST passive flotation did not. All these 26 samples that showed discrepant results contained < 50 eggs per gram (epg) by either method. Agreement of the two centrifugal flotation techniques was 94.5% across the four targeted parasites. In this evaluation VETSCAN IMAGYST centrifugal flotation detected 5 samples that conventional centrifugal flotation did not; conventional centrifugal flotation detected 12 that VETSCAN IMAGYST centrifugal flotation did not. Of these 17 discrepant results, 13 contained < 50 epg by either method.

**Table 2:** Diagnostic sensitivity and specificity comparison of samples prepared with VETSCAN IMAGYST passive flotation *versus* OVASSAY passive flotation (reference method), both read by a parasitologist

	*Ancylostoma*	*Trichuris*	*Toxocara*	Taeniidae
True positive	40	22	21	4
False positive	4	6	0	6
True negative	53	67	78	90
False negative	3	5	1	0
Total	100	100	100	100
Sensitivity (%) (95% CI)	93.0 (82.5–98.0)	81.5 (64.1–92.6)	95.5 (80.7–99.5)	100 (55.5–100)
Specificity (%) (95% CI)	93.0 (84.2–97.6)	91.8 (83.8–96.5)	100 (96.8–100)	93.8 (87.6–97.3)

**Table 3:** Diagnostic sensitivity and specificity comparison of samples prepared with VETSCAN IMAGYST centrifugal flotation *versus* conventional centrifugal flotation (reference method), both read by a parasitologist

	*Ancylostoma*	*Trichuris*	*Toxocara*	Taeniidae
True positive	42	25	20	10
False positive	2	3	1	0
True negative	53	64	76	88
False negative	3	8	3	2
Total	100	100	100	100
Sensitivity (%) (95% CI)	93.3 (83.3–98.1)	75.8 (59.4–87.8)	87.0 (69.1–96.2)	83.3 (56.4–96.4)
Specificity (%) (95% CI)	96.4 (88.8–99.2)	95.5 (88.5–98.7)	98.7 (94.1–99.9)	100 (97.2–100)

*Abbreviation*: CI, confidence interval

As a framework for comparison, Table 4 summarizes the performance of conventional centrifugal flotation *versus* OVASSAY passive flotation. These two standard fecal sample preparation methods showed comparable or lower correlation to one another than the VETSCAN IMAGYST methods showed to conventional methods. Agreement of OVASSAY passive flotation and standard centrifugal flotation was 94.3%. OVASSAY detected 4 samples that standard centrifugal flotation did not, while standard centrifugal flotation detected 19 samples that OVASSAY did not. Of these 23 discrepant results, 15 contained < 50 epg by either method.

**Table 4:** Diagnostic sensitivity and specificity comparison of samples prepared with conventional centrifugal flotation *versus* OVASSAY passive flotation, both read by a parasitologist

	*Ancylostoma*	*Trichuris*	*Toxocara*	Taeniidae
True positive	41	26	22	4
False positive	2	1	0	0
True negative	53	66	77	88
False negative	4	7	1	8
Total	100	100	100	100
Sensitivity (%) (95% CI)	91.1 (80.2–96.9)	78.8 (62.8–90.0)	95.7 (81.4–99.5)	33.3 (12.5–61.2)
Specificity (%) (95% CI)	96.4 (88.8–99.2)	98.5 (93.2–99.8)	100 (96.8–100)	100 (97.2–100)

*Abbreviation*: CI, confidence interval

### System performance

The workflow, combining image analysis performed by deep learning object detection algorithm with the specialized fecal sample preparations, provided a fecal examination result with images within 10–14 min, depending on the fecal sample preparation method chosen (Table 5).

**Table 5:** Time-to-result of the full VETSCAN IMAGYST system including sample preparation, slide scan, and image analysis

	Passive flotation	Centrifugal flotation
Scan time	6.6 min ± 37 s (*n* = 99)	6.8 min ± 49 s (*n* = 100)
Total time to result^a^	14 min ± 73 s (*n* = 9)	10.3 min ± 65 s (*n* = 7)

^a^Preparation, scan and analysis

## Discussion

To the best of our knowledge, this is the first study demonstrating that a deep learning object detection algorithm successfully recognized and identified intestinal parasite eggs of dogs and cats on fecal flotation slides scanned by an automated microscope. The utilization of machine learning systems to support veterinarians with decision and diagnosis making processes has been evaluated previously [12–17]; however, it has been very limited in veterinary medicine compared to that in human medicine [18]. The algorithms evaluated in previous studies were mainly systems that assisted the medical decision-making processes based on results obtained from physical examinations and laboratory tests [12–17]. Additionally, a computational shape recognition system integrated with fluorescent lebelling and smartphone-based image capturing has been assessed and applied for parasite fecal egg counting examinations [19, 20].

A recent study applied a computer vision plus a support vector machine for detection of canine intestinal parasites and evaluated its capability with *Ancylostoma* eggs, *Toxocara* eggs, *Trichuris* eggs and *Giardia* cysts [21]. In the first step of their process, images were segmented into blobs using a combination of color thresholding and other computer vision techniques. Since this process relies heavily on manual experimentation, it is limited by the ability and creativity of a developer to program how the image can be divided to detect objects of interest. In addition, the process could be brittle because computer vision methods can become completely unusable with minor variations in white balance, brightness, stains, and/or other factors which are easily compensated by the human eye. In the second step, features were manually selected based upon object area, color, aspect ratio, etc., to allow a classifier to use those visual features. Manual feature selection, however, can easily become biased and cause issues because it is difficult for humans to represent visual features numerically and features chosen by humans are sometimes based on their ease and intuition rather than what is most discriminative for a classifier. Finally, the selected features were used to train a support vector machine. Although the support vector machine can automatically learn from new data, this process does not always generalize well because it is entirely dependent on the previous steps, which can be brittle due to reliance on manual inputs.

In contrast, the VETSCAN IMAGYST applies a deep learning object detection algorithm which uses convolutional layers that automatically learn the most discriminating features between classes. This method has been shown to be significantly more accurate than previous conventional, shallow learning systems [22]. The deep learning system is also more robust because it can learn to ignore the features that make computer vision brittle. Furthermore, learned features automatically generalize to new data and become available to allow new classes to be learned with fewer examples [23]. In addition to the deep learning aspect, the VETSCAN IMAGYST utilizes an SSD object detection model with an Inception v2 backbone that combines localization and classification as a single regression problem [9, 10]. With this model, learned features can be reused for both localizing and classifying steps, which results in faster training and inference because the majority of the processing time is utilized encoding features. This allowed the VETSCAN IMAGYST to perceive and distinguish the morphology of parasite eggs from other objects on fecal flotation slides in less than seven minutes. Moreover, sharing localization and classification features allows the system to use contextual information aiding in classification which has been shown to decrease the number of background errors [9, 10]. Overall, two main characteristics, the deep learning and combination of localization and classification systems, led to the agreeable results on comparison between the VETSCAN IMAGYST system and parasitologists’ examinations within a feasible processing time (Fig. 3, Tables 1, 5).

Although the ability of the VETSCAN IMAGYST system keeps improving as previously mentioned, there were some limitations. The system did not examine on the border or outside of a coverslip due to the defined scan area. Occasionally, parasite eggs were observed microscopically at the edge or outside of a coverslip by a parasitologist’s examination especially when a larger amount of the solution was placed on a fecal slide; however, these eggs were not recognized by the VETSCAN IMAGYST system. As a common limitation for many object detection models [24], the VETSCAN IMAGYST system struggled to precisely localize and distinguish small objects, though further algorithm improvements can ameliorate this limitation.

The VETSCAN IMAGYST passive and centrifugal fecal flotation techniques exhibited equivalent qualitative identification of the targeted parasite eggs compared to the reference passive and centrifugal fecal flotation techniques (Tables 2, 3). The discrepancies observed in 26 fecal samples by passive flotation methods and in 17 fecal samples by centrifugal flotation methods were most likely due to the inherent subsampling variability in non-homogenous fecal samples that has been well documented in previous publications [8, 25]. As Kochanowski et al. [25] demonstrated a wide range of coefficients of variation (31–254%) in *Toxocara* and *Trichuris* samples of 50 epg and lower, 39 of the 43 discrepant samples observed in the present study contained < 50 epg, and the remaining samples contained a medium number of parasite eggs. In addition to the subsampling variability, a variation of fecal samples, such as consistency (liquid *vs* dry) and amounts of debris (grasses, pebbles, etc.) included, could have influenced the inconsistent results since only 1 gram of feces was utilized for each technique. A low number of fecal samples, especially samples of *Trichuris*, *Toxocara* and Taeniidae, also limited the evaluation of the diagnostic sensitivity and specificity of the VETSCAN IMAGYST sample preparation techniques. Due to the small sample size, analysis of the accuracy and precision of the techniques was not performed.

The sample preparation time using the VETSCAN IMAGYST device was approximately 3.5 minutes with a 2-minute centrifugal incubation time and 7.4 minutes with a 5-minute passive incubation time (Table 5), which is comparable or even quicker than the preparation time for conventional fecal flotation tests. The VETSCAN IMAGYST device is flexible and can be utilized with either a centrifugal or passive fecal flotation process, although centrifugation significantly increases the sensitivity of the fecal examinations (Table 4).

The present study also demonstrated that centrifugation significantly increases sensitivity of fecal examination (Table 4), which is consistent with previous studies [1, 2, 4–6, 8, 26]. Compared to centrifugal flotation, passive flotation is known to be less reliable to recover *Trichuris vulpis* eggs, *Physaloptera* eggs, tapeworm eggs/egg packets, *Giardia* cysts, and any forms of parasite present in low numbers in feces [1, 2, 4–6, 8, 26], even though passive flotation is still more commonly used in private practices for its convenience. If passive flotation is the only fecal examination performed at a veterinary practice, veterinarians need to remember it reduces the sensitivity and may need to repeat a test multiple times or refer to a reference diagnostic laboratory where more sensitive tests, including a centrifugal fecal flotation test, are offered.

The deep learning nature of the algorithms used for the present analysis will allow for improved performance and functionality over time. With more training, the algorithm will be able to distinguish other parasites, eggs, oocysts, cysts, and trophozoites, besides the targeted parasite eggs included in the present study. In addition, the algorithmic screening system was able to estimate the number of targeted parasite eggs simultaneously, though this was not analyzed in the present study. This capability will be developed further in the near future, and it is anticipated the algorithm will be able to perform a fecal egg/oocyst counting test. Since all captured photographs are stored in the cloud, any images with suspicious objects captured by image analysis can be emailed and evaluated by parasitologists at any time, and the parasite photographs can also be used as teaching and training materials for new staff, veterinary students, and technicians.

## Conclusions

The VETSCAN IMAGYST scanning and analyzing systems with the VETSCAN IMAGYST fecal preparation techniques reliably detected *Ancylostoma*, *Toxocara*, *Trichuris vulpis* and taeniid eggs in feces of dogs and cats and enabled completion of a fecal examination, from fecal preparation to obtaining a result, within 15 minutes, without the need for trained expertise. As the VETSCAN IMAGYST algorithm improves and learns additional parasites, this system can be used at veterinary clinics assisting veterinarians and technicians to perform routine fecal flotation tests accurately and efficiently.

## Data Availability

All data generated or analyzed during this study are included in this published article.

## Abbreviations

SSD: single shot MultiBox detector
epg: eggs per gram
